# Association of *HMGB1 *polymorphisms with outcome in patients with systemic inflammatory response syndrome

**DOI:** 10.1186/cc6935

**Published:** 2008-06-24

**Authors:** Brian Kornblit, Lea Munthe-Fog, Hans O Madsen, Jens Strøm, Lars Vindeløv, Peter Garred

**Affiliations:** 1 Department of Clinical Immunology – 7631, Rigshospitalet, University of Copenhagen, Blegdamsvej 9, 2100 Copenhagen O, Denmark; 2 The Allogeneic Hematopoietic Cell Transplantation Laboratory – 4041, Department of Haematology, Rigshospitalet, University of Copenhagen, 2100 Copenhagen O, Denmark; 3 Department of Research, Sygehus Sønderjylland, University of Southern Denmark, 6200 Aabenraa, Denmark

## Abstract

**Introduction:**

High mobility group box 1 protein (HMGB1) is a pleiotropic cytokine, recently implicated in the pathophysiology of the systemic inflammatory response syndrome (SIRS) and sepsis. Data from experimental sepsis models show that administration of anti-HMGB1 antibodies significantly decreased mortality, even when administration was delayed for 24 hours, providing a window of opportunity for therapeutic intervention if transferred into a clinical setting. Whether genetic variation in the human *HMGB1 *gene is associated with disease susceptibility is unknown.

**Methods:**

We sequenced the *HMGB1 *gene in 239 prospectively monitored patients with SIRS admitted to an intensive care unit and we measured the corresponding HMGB1 serum concentrations. Blood donors served as control individuals. Outcome parameters according to different *HMGB1 *genotypes were compared.

**Results:**

Homozygosity and heterozygosity for a promoter variant (-1377delA) was associated with a decreased overall 4-year survival (15% versus 44%, hazard ratio = 1.80; *P *= 0.01) and with a decreased number of SIRS criteria. Carriage of an exon 4 variant (982C>T) was significantly associated with an increased number of SIRS criteria, a higher Simplified Acute Physiology Score II score, a lower PaO_2_/FiO_2 _ratio and lower serum HMGB1 levels (*P *= 0.01), and with a significantly higher probability of early death due to infection (*P *= 0.04). HMGB1 was undetectable in the control individuals.

**Conclusion:**

The present article is the first report of clinical implications of variation in the human *HMGB1 *gene. Two polymorphisms were determined as significant risk factors associated with early and late mortality, which may provide insight into the molecular background of SIRS and sepsis, suggesting a possible role for HMGB1 genetics in future prognostic evaluation.

## Introduction

The systemic inflammatory response syndrome (SIRS), which is the most common cause of death in intensive care units (ICUs) [1], was formally defined in 1992 [2] as a clinical syndrome referring to the effects of severe systemic inflammation associated with both infectious and noninfectious etiologies [3]. The pathophysiology of SIRS is complex, with factors related to the initial etiological event and the host's immune system playing a role in determining the outcome (reviewed in [4]). Why some patients die while other patients survive similar insults is partly unknown, but may to some extent be explained by the genetic variation. In recent years several studies have been published showing the importance of variation in cytokine genes on the inflammatory response affecting individual susceptibility to sepsis, risk of complications and death (reviewed in [5]). The effect of genetic variation is an area of intensive debate due to conflicting results [5].

High mobility group box 1 protein (HMGB1) is a highly conserved [6] and ubiquitously expressed protein [7], originally discovered as a nonhistonal nuclear DNA binding protein [8]. The essential importance of HMGB1 as a pleiotropic cytokine became apparent in a series of experiments showing that HMGB1 was actively secreted from monocytes and macrophages in response to challenges with lipopolysaccharide and by a TNFα-dependent mechanism [9]. Furthermore, the study found that HMGB1 levels were significantly elevated in patients who succumbed to sepsis, and that the administration of HMGB1 in murine models caused sepsis-like symptoms and death [9]. In experimental murine models of endotoxemia [9] and sepsis, administration of anti-HMGB1 antibodies decreased mortality significantly [9,10], even when administration was delayed for 24 hours [10], providing a window for therapeutic intervention if transferred into a clinical setting.

These experiments showed that HMGB1 – in contrast to other inflammatory cytokines, such as TNFα – is a late mediator of inflammation. Besides being actively secreted, HMGB1 is passively released from necrotic cells but not from apoptotic cells, creating a signal for the organism to distinguish between these two types of cell death [11]. Several studies have now been published on HMGB1 and infection, and the general consensus is that HMGB1 levels are increased in patients with sepsis as compared with healthy control individuals [9,12-17]. There is, however, evidence that HMGB1 is also involved in the pathophysiology of a variety of other diseases with no obvious infectious etiology: rheumatoid arthritis [18,19], hemorrhagic shock [20], cerebral and myocardial ischemia [21], acute lung injury [14] and acute pancreatitis [22].

The human *HMGB1 *gene is located on chromosome 13, and six polymorphic loci throughout the gene locus have recently been identified [23]. In the present study we asked whether *HMGB1 *variant alleles are associated with serum HMGB1 levels and outcome in patients with SIRS admitted to the ICU.

## Materials and methods

### Patients

The present study was based upon a previously published cohort of consecutive patients > 17 years old, admitted to the Intensive Care Unit, Glostrup University Hospital, Copenhagen, Denmark, who met the criteria for SIRS [24]. Patients were assessed for Simplified Acute Physiology Score II (SAPS II) [25] within 24 hours of admission to the ICU, and the highest score was recorded. Peripheral blood samples for serum measurements and DNA extraction were obtained immediately after admission. Samples from 239 patients were eligible for this study. Patients were excluded if the neutrophil count was < 1.0 × 10^9 ^cells/l before the onset of sepsis, if the infection was associated with burns, if the patient had suspected or documented recent acute myocardial infarction, or if there was a lack of commitment to full life-support measures by the primary physician. Information about death during follow-up was obtained from the Danish Central Office of Civil Registration.

The control population consisted of 103 healthy Danish Caucasian blood donors, and has previously been published [23]. Furthermore, HMGB1 serum concentrations were measured in 20 healthy Danish Caucasian blood donors.

Informed consent was obtained from all patients or from their close relatives. The study was approved by the local ethics committee.

### Classification criteria for SIRS, sepsis, severe sepsis and septic shock

SIRS was defined as outlined by the American college of Chest Physicians/Society of Critical Care Medicine Consensus Conference [2]. Sepsis was defined as SIRS with a documented or clinically suspected infection, while severe sepsis was defined as the presence of sepsis with at least one of the following organ dysfunction criteria developing within 24 hours of enrolment in the study: arterial systolic blood pressure < 90 mmHg for at least 1 hour, despite appropriate fluid resuscitation or vasopressor therapy; urine output < 0.5 ml/kg for > 1 hour, despite hydration; PaO_2_/FiO_2 _ratio ≤ 40 kPa; acute deterioration of mental status (Glasgow Coma Score < 14); unexplained metabolic acidosis with pH ≤ 7.30 or base deficit ≥ 5.0 mM in association with plasma lactate ≥ 1.6 mM; or hepatobilliary dysfunction with serum bilirubin > 34 μM and no evidence of pre-existing hepatobilliary disease. Septic shock was defined as sepsis with hypotension and one of the above listed organ dysfunction criteria.

### Genotyping

Reference single nucleotide polymorphism numbers (rs) are provided for genetic variants. All PCR and sequencing primers were synthesised by DNA-Technology A/S (Aarhus, Denmark). The DNA sequence containing the -1615A>G (rs1412125) and -1377delA (rs41369348) genetic variants was amplified using primer set A; the sequence containing the 1747delT (rs55946320), 1779 T>G (rs41433050), 1808C>G (rs55832802), 1822G>A (rs41534245) and 1888insT (rs41497949) variants was amplified using primer set B; and the 2351insT (rs41376448) and 4519_4521delGAT (rs56178645) variants were amplified using primer set C (Table 1). Forward primers contained a 5'-T7 tag (5'-TAA TAC GAC TCA CTA TAG GG-3').

**Table 1:** PCR and pyrosequencing primers

Genetic variant	PCR			Pyrosequencing primer
		
	Primer set	Forward primer	Reverse primer	
Sequencing				
-1615A>G	A	5'-ATG TGC ATG TGT GAT ATA TTG TCC-3'	5'-GTT ATA TCA GTG CTT TAT GAA ACT AC-3'	
-1377delA				
1747delT	B	5'-CAA AGT TTT ATG CAA GGA GGG TG-3'	5'-GTC CAT TCA GGG CGA TCT C-3'	
1779T>G				
1808C>G				
1822G>A				
1888insT				
4519_4521delGAT	C	5'-AAA GTT CTG CCA TGT TCT ATT TC-3'	5'-CAG GAC AGG GCT ATC TAA AG-3'	
2351insT				
Pyrosequencing				
-196C>A		5'-CTC TTT GCC CGG CAT ACA CA-3'	5'-TCC TGA CCA GAG CCC GTT T-3'	5'-TTG ATG ACG TGT CCC-3'
3814C>G		5'-GTC TGA TTT TAC GGA GGT TGA-3'	5'-CCT TTG CCC ATG TTT AGT TAT T-3'	5'-TAC TTT GGT TTT CAT TCC-3'
982C>T		5'-TGT TCA TCT AGG GTT CTA GCT-3'	5'-CCT TTG ATT TTT GGG CGA TAC-3'	5'-TTA GTT CGG CCT TCT T-3'
1177G>C		5'-AAC TGG GAG AGA TGT GGA ATA-3'	5'-CAA TCA TAC ATC TGG CGT ACT-3'	5'-GGT TTG CTT GGT AAA ATG-3'

Forward primers from primer sets A, B and C contain a 5'-T7 sequence (5'-TAA TAC GAC TCA CTA TAG GG-3'). The 5' ends of all of the PCR reverse primers used for pyrosequencing are biotinylated.

PCR amplification was performed in a volume of 12 μl, containing 0.25 μM forward and reverse primers, 0.50 μl DNA, 2 mM MgCl_2_, 0.27 mM dNTP, 67 mM KCl, 27 mM Tris-HCl, pH 8.4, and 0.03 U Platinum Taq DNA polymerase (Invitrogen Corp., Carlsbad, CA, USA). Amplification was carried out on a GeneAmp PCR System 9700 (Applied Biosystems, Foster City, CA, USA) under the following cycling conditions: 94°C for 2 minutes; 35 cycles of 94°C for 30 seconds, 60°C for 1 minute and 72°C for 1 minute; and 72°C for 5 minutes. The PCR products were sequenced using the ABI BigDye cycle sequencing terminator kit (Applied Biosystems) according to the manufacturer's protocol.

The DNA regions containing the -1615 A>G, -1377delA, 4519_4521delGAT and 2351insT variants were sequenced in the forward direction with 0.16 μM biotin-conjugated T7 sequence primers, and were subsequently purified using streptavidin sepharose beads. The DNA region containing the 1747delT, 1779T>G, 1808C>G, 1822G>A and 1888insT variants was sequenced in the reverse direction with 0.16 μM primer set B reverse primer and was purified by ethanol precipitation preceded by ExoSAP-IT (USB Corp., Cleveland, OH, USA) clean up, according to the manufacturer's protocol.

Streptavidin sepharose high-performance bead (GE healthcare Bio-Sciences Corp., Piscataway, NJ, USA) purification was performed by incubating 2.5 μl PCR product with 3 μl beads and 25 μl binding buffer (3 mM Tris-HCl, pH 7.5, 0.3 mM ethylenediamine tetraacetic acid, 0.6 M NaCl) for 5 minutes under continuous agitation. The beads were hereafter picked up by the Vacuum Prep Tool (Biotage AB, Uppsala, Sweden) and were rinsed by sequential 10-second aspirations of 70% ethanol, 0.2 M NaOH and 70% ethanol, followed by release into 15 μl deionised formamide (Amresco, Solon, OH, USA).

Ethanol precipitation was performed by adding 96% EtOH with 25 μl of 0.1 M NaOAc (pH 4.6) to 10 μl sample, with subsequent centrifugation for 30 minutes at 2,500 × *g *and for 1 minute inverted at 150 × *g*. Thereafter, 50 μl of 70% EtOH was added and the sample was centrifuged for 5 minutes at 2,500 *g *and for 1 minute inverted at 150 × *g*. Finally, 15 μl deionised formamide was added.

After purification, the sequence analysis was performed on an ABI Prism 3100 Genetic Analyzer (Applied Biosystems). The DNA sequences were aligned using BioEdit software version 7.0.5.3 [26] and polymorphisms were confirmed visually from sequence electropherograms. New genetic variants were confirmed by reverse sequencing and were submitted to the dbSNP database [27].

The -196C>A (rs41477046), 982C>T (rs1060348), 1177G>C (rs3742305) and 3814C>G (rs2249825) variants were genotyped by using the PSQ 96 MA pyrosequencing platform (Biotage AB) according to the manufacturer's protocols. PCR was performed in a volume of 30 μl under the same conditions as described above. The PCR and pyrosequencing primers are presented in Table 1.

### Serum high mobility group box 1 protein

HMGB1 serum concentrations were measured using the HMGB1 ELISA Kit II (Shino-Test Corporation, Tokyo, Japan) according to the manufacturer's protocols.

### Statistical analysis

The inferred haplotypes and linkage disequilibrium, expressed as *D' Lewontin's coefficient *and the squared correlation coefficient *R*^2 ^quantified betweenall pairs of biallelic loci, were estimated using the SNPAlyzeprogram version 4.0 (Dynacom, Yokohama, Japan). The Hardy-Weinberg equilibriumwas analysed using gene frequencies obtained by simple genecounting and the chi-square test with Yates' correction forcomparing observed and expected values. Fisher's exact test was used to compare frequencies, and the Wilcoxon test and the Kruskall-Wallis test were used to compare continuous data. Survival was estimated by the Kaplan-Meier method, and comparisons were made with the log-rank test. Cumulative incidence estimates were calculated to allow for death from causes other than infection to be treated as a competing risk [28], and curves were compared using Gray's *k*-sample test [29]. Cox regression analysis was performed when appropriate. All *P *values were two-sided and *P *< 0.05 was considered significant.

## Results

### Genetic variation in the *HMGB1 *gene

Table 2 presents the frequency of the polymorphisms in the *HMGB1 *gene in 239 SIRS patients and in 103 healthy control individuals. There was no significant difference between the SIRS patients and the control population in the frequency of polymorphisms (*P *> 0.05).

**Table 2:** Distribution of genotypes

Polymorphism	rs number	Control population			SIRS population		
-1615A>G	1412125	A/A	A/G	G/G	A/A	A/G	G/G
		24.0	47.0	29.0	24.0	48.0	28.0
-1377delA	41369348	A/A	A/-	-/-	A/A	A/-	-/-
		87.0	13.0	0.0	89.5	10.0	0.5
1747delT	55946320	T/T	T/-	-/-	T/T	T/-	-/-
		96.0	4.0	0.0	97.0	3.0	0.0
1888insT	41497949	-/-	-/T	T/T	-/-	-/T	T/T
		99.0	1.0	0.0	99.0	1.0	0.0
3814C>G	2249825	C/C	C/G	G/G	C/C	C/G	G/G
		47.0	47.0	6.0	53.0	41.0	6.0
982C>T	1060348	C/C	C/T	T/T	C/C	C/T	T/T
		92.0	8.0	0.0	95.0	5.0	0.0
1177G>C	3742305	G/G	G/C	C/C	G/G	G/C	C/C
		47.0	46.0	7.0	53.0	41.0	6.0
2351insT	41376448	-/-	-/T	T/T	-/-	-/T	T/T
		50.0	45.0	5.0	57.0	38.0	5.0

Observed frequencies (%) of polymorphisms in 103 healthy Danish Caucasian control individuals and in 239 systemic inflammatory response syndrome (SIRS) patients (*P *> 0.05). rs number, reference single nucleotide polymorphism number.

**Figure 1:**
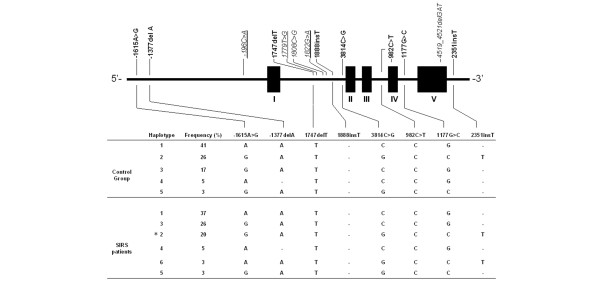
Schematic illustration of the high mobility group box 1 protein gene locus. The most common inferred haplotypes (frequency > 3%) in both the control population and the systemic inflammatory response syndrome (SIRS) population are shown. Modified from Ferrari and colleagues [41]. Bold, location of polymorphisms; italic, location of mutations; underline, mutations only found in the control population; solid boxes, exons I to V. *Haplotype was statistically more frequent in the SIRS population (*P *= 0.006) as compared with the control population.

The -196C>A, 1779T>G and 1822insT mutations were not observed in the SIRS patients, while the 1888insT mutation was observed in two SIRS patients and was therefore treated as a polymorphism, although not meeting the formal requirement of an allele frequency ≥ 1%. The novel polymorphism 1747delT, entailing a deletion of a single thymine in a sequence of 11 thymine nucleotides in intron 1 (Figure 1), was observed in the SIRS patients. The control subjects were reanalysed and, upon reverse sequencing, four individuals were found heterozygous for 1747delT. Two novel genetic variants, 1808C>G and 4519_4521delGAT, were identified once in two different SIRS patients, and were therefore classified as mutations. The 4519_4521delGAT entailed a deletion of the trinucleotide sequence GAT in exon 5 (Figure 1), leading to deletion of an aspartate amino acid from the acidic tail of the HMGB1 protein.

All polymorphisms adhered to the Hardy-Weinberg equilibrium expectations (*P *< 0.05). The pairwise investigation between the eight polymorphic loci indicated strong linkage disequilibrium between several genotype variants (Table 3). Only genetic variants defined as polymorphisms were used in the construction of haplotypes. Out of 43 and 45 possible inferred haplotypes in the SIRS and control populations, respectively, the polymorphisms only segregated as 13 and 15 distinct haplotypes. The most common haplotypes (frequency > 3%) in the control and SIRS populations are shown in Figure 1. Observed haplotype frequencies were similar in both populations – except for haplotype 2, which was observed with a significantly (*P *= 0.006) higher frequency in the control population than in the SIRS patients.

**Table 3:** Pairwise linkage disequilibrium (expressed by *D' Lewontin's coefficient*/squared correlation coefficient *R*^2^) between polymorphisms

	-1615A>G	-1377delA	1747delT	1888insT	3814C>G	982C>T	1177G>C
-1377delA	1/0.062						
1747delA	1/0.0137	0.0658/0.0010					
1888insT	1/0.00453	0.4401/0.0141	-1/0.00005				
3814C>G	-0.7193/0.1736	-1/0.0208	-1/0.0046	-0.1868/0.00005			
982C>T	-1/0.0239	-1/0.00148	-1/0.000327	-1/0.0001	-0.7274/0.00493		
1177G>C	-0.7234/0.1775	-1/0.021	-1/0.00465	-0.2114/0.00007	1/0.9894	-0.2228/0.00047	
2351insT	-0.6433/0.1202	-1/0.018	-1/0.00398	0.0443/0.00003	0.9503/0.7816	0.0462/0.00018	0.9626/0.7935

### Patient characteristics

Out of the 239 patients with SIRS who were enrolled in this study, 63 patients had SIRS without infection and 24 patients fulfilled the criteria for sepsis, 87 patients for severe sepsis and 65 patients for septic shock. The median follow-up was 698 days (range, 1 to 1,458 days), with an overall survival of 41%. Patients were admitted to the ICU for a median of 3 days (range, 0 to 38 days) and 48 patients died while in the ICU. There were no significant differences in the distribution of polymorphisms between the SIRS without infection group, the sepsis group, the severe sepsis group and the septic shock group. Baseline characteristics and overall survival probabilities are outlined according to admission status in Table 4.

**Table 4:** Patient baseline characteristics and mortality according to admission status

Variable	SIRS without infection group	Sepsis group	Severe sepsis group	Septic shock group
Number of patients (%)	63 (26.4%)	24 (10.0%)	87 (36.4%)	65 (27.2%)
Age (years)	58.5 ± 18.2	54.8 ± 15.4	63.1 ± 14.9	64.1 ± 15.1
Type of admission				
Acute surgery	33 (13.8)	11 (4.6)	32 (13.4)	26 (10.9)
Elective surgery	4 (1.7)	1 (0.4)	2 (0.8)	0 (0)
Medical	26 (10.9)	12 (5.0)	53 (22.2)	39 (16.3)
Chronic disease				
Metastatic cancer	0	0	0	1
Hematological malignancy	1	0	2	0
SAPS II score^a^	32.7 ± 15.8	23.4 ± 8.4	40.4 ± 12.1	45.7 ± 14.4
PaO_2_/FiO_2 _ratio^a^	183 ± 93	351 ± 53	152 ± 89	137 ± 82
Serum HMGB1 (ng/μl)	0.94 ± 1.81	1.49 ± 3.15	1.38 ± 2.73	1.79 ± 4.00
Number of SIRS criteria^a^				
2	31 (13.0)	8 (3.3)	17 (7.1)	5 (2.1)
3	20 (8.4)	8 (3.3)	31 (13.0)	19 (7.9)
4	12 (5.0)	8 (3.3)	39 (16.3)	41 (17.2)
Overall survival (%)				
Day 28	87	88	62	65
Follow-up time	52	71	25	41

Data presented as *n *(%), mean ± standard deviation or percentage. ^a^Comparison of systemic inflammatory response syndrome (SIRS) without infection versus sepsis, severe sepsis and shock, and comparison of sepsis versus severe sepsis versus septic shock, *P *< 0.01. HMGB1, high mobility group box 1 protein; PaO_2_/FiO_2 _= ratio between partial pressure of oxygen and fraction of inspired oxygen; SAPS II, Simplified Acute Physiology Score II.

### Influence of *HMGB1 *genotype on survival

**Figure 2:**
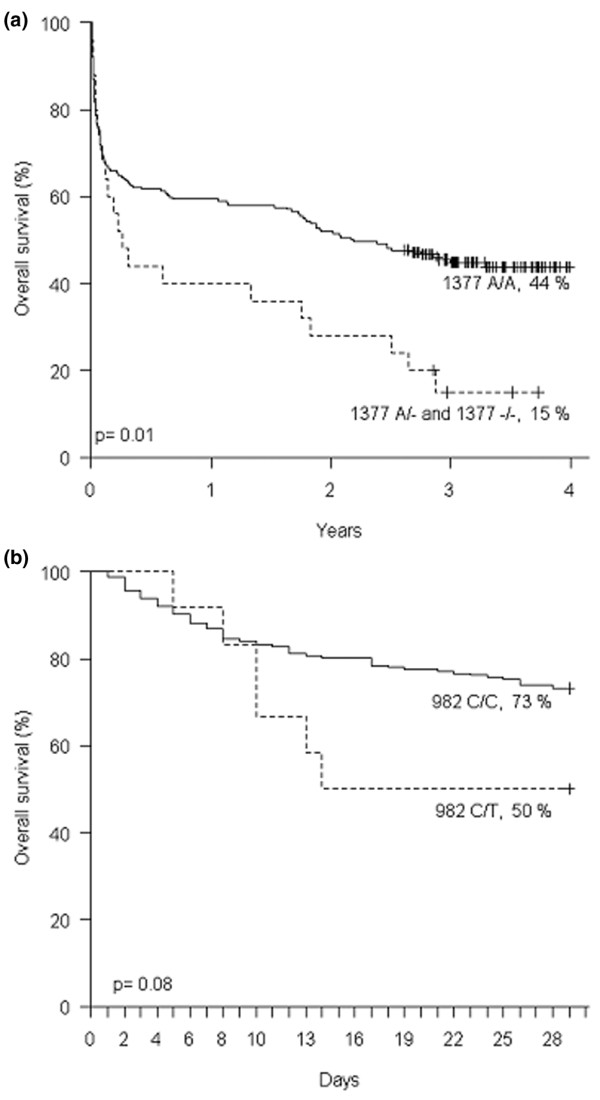
Influence of *HMGB1 *genotype on survival. **(a) **Overall survival during the follow-up period according to the -1377delA genotype. **(b) **28-day survival according to the 982C>T genotype.

Stratification of the patients according to *HMGB1 *genotype revealed a significantly lower overall survival in patients with the -1377delA^A/- ^or -1377delA^-/- ^genotypes as compared with patients with the -1377delA^A/A ^genotype (15% versus 44%, *P *= 0.01) (Figure 2a). For statistical purposes, the one patient homozygous for -1377delA^-/- ^was included in the -1377delA^A/- ^group in all analyses. The decreased overall survival in patients with the -1377delA^A/- ^or -1377delA^-/- ^genotypes was discernible in the survival curves after approximately day 50 (Figure 2a), but did not become significant until the end of follow-up. In Cox regression analysis, the -1377delA^A/- ^or -1377delA^-/- ^genotype was an independent risk factor significantly associated with death, in both a restricted survival model and in an expanded model, including known factors associated with survival (age and number of SIRS criteria) (Table 5).

**Table 5:** Cox regression survival models

	Hazard ratio	95% confidence interval	*P *value
Restricted model			
-1377delA genotype	1.80	1.13 to 2.87	0.01
Expanded model			
-1377delA genotype	1.84	1.14 to 2.96	0.028
Age	1.04	1.03 to 1.05	< 0.001
Number of SIRS criteria	1.42	1.13 to 1.80	0.003

SIRS, systemic inflammatory response syndrome.

Although no other genotypes were associated with differences in survival, patients with the 982C/T genotype had near-significant (*P *= 0.08) decreased day 28 survival as compared with patients with the 982C/C genotype (Figure 2b). In order to allow for the presence of competing risks, the cumulative incidence was calculated. This incidence revealed a significantly (*P *= 0.04) higher probability of death due to infection in patients with the 982C/T genotype as compared with patients with the 982C/C genotype (50% versus 20%).

There were no significant differences in age, gender and type of admission between patients in the -1377delA^A/- ^group, the -1377delA^-/- ^group or the 982C/T group and the rest of the cohort. Haplotypes one to six from the SIRS population were not associated, independent of the -1377delA^A/- ^or -1377delA^-/- ^and 982C/T genotypes, with any disease parameters or outcomes.

### Serum HMGB1 levels, SIRS criteria, SAPS II score and PaO_2_/FiO_2 _ratio

The serum HMGB1 levels in the 20 healthy control individuals were all below the detection level of the assay (0.3125 ng/μl), and were significantly different (*P *< 0.001) from the mean serum HMGB1 level of the patient population. The mean serum HMGB1 levels tended to increase when moving from patients with only SIRS to patients with sepsis of increasing severity, although only reaching a near-significant level (*P *= 0.08) when comparing SIRS patients with sepsis patients regardless of severity (Table 4). No significant difference in serum HMGB1 levels was seen between survivors and nonsurvivors (*P *= 0.13). There was no correlation between serum HMGB1 levels and the number of SIRS criteria, the SAPS II score or the PaO_2_/FiO_2 _ratio.

The serum HMGB1 levels in patients with the 982C/T genotype were significantly lower (*P *= 0.008) as compared with patients with the C/C genotype, but were not different from healthy control individuals (*P *= 0.19). No significant difference (*P *= 0.37) was found between patients with the -1377delA^A/A ^genotype and the -1377delA^A/- ^or -1377delA^-/- ^genotype (Table 6). Patients with the 982C/T genotype had a higher number of SIRS criteria (*P *= 0.002), a lower PaO_2_/FiO_2 _ratio (*P *= 0.003), and a tendency towards a higher SAPS II score (*P *= 0.108), as compared with patients with the C/C genotype. Patients with the -1377delA^A/- ^or -1377delA^-/- ^genotype had a lower number of SIRS criteria (*P *= 0.008), a tendency towards a lower PaO_2_/FiO_2 _ratio (*P *= 0.07), and no difference in SAPS II score (*P *= 0.73), as compared with patients with the -1377delA^A/A ^genotype (Table 6).

**Table 6:** Classification of serum HMGB1, number of SIRS criteria, SAPS II score and PaO_2_/FiO_2 _ratio according to the *HMGB1 *982C>T and -1377delA genotypes

Variable	982C>T genotype		-1377delA genotype	
	
	C/C	C/T	A/A	A/- or -/-
Serum HMGB1 (ng/μl)	1.45 ± 3.05	0.28 ± 0.96*	1.36 ± 2.78	1.61 ± 4.43
Number of SIRS criteria	3.13 ± 0.81	3.83 ± 0.39*	3.21 ± 0.80	2.76 ± 0.78*
SAPS II	37.8 ± 15.2	43.9 ± 12.3	38.2 ± 15.3	36.8 ± 13.8
PaO_2_/FiO_2 _ratio	164 ± 94	89 ± 38*	158 ± 96	182 ± 63

Data presented as mean ± standard deviation. *P = 0.01. HMGB1, high mobility group box 1 protein; PaO_2_/FiO_2 _= ratio between partial pressure of oxygen and fraction of inspired oxygen; SAPS II, Simplified Acute Physiology score II; SIRS, systemic inflammatory response syndrome.

## Discussion

The pathophysiology of SIRS and sepis is complex, and the recognition that inherited traits influence individuals' susceptibility and ability to respond appropriately to inflammation [30] has prompted several studies exploring the association between genotypes and outcome. Polymorphisms in innate immune receptors and in both proinflammatory and anti-inflammatory cytokines have been extensively studied in relation to SIRS and sepsis (reviewed in [5]).

In the present study, several associations were observed revealing the importance of the genetic variation in the *HMGB1 *gene. The -1377delA^A/- ^genotype or the -1377delA^-/- ^genotype showed a significant association with delayed mortality, independent of age and number of SIRS criteria. In agreement with this, a significant association with lower number of SIRS criteria and a tendency to a higher PaO_2_/FiO_2 _ratio was observed, predicting better early survival. The impact of the -1377delA polymorphism on mortality was already discernable at approximately day 50 after admission, suggesting that the initial insult related to the ICU admission renders the patients carrying the -1377delA polymorphism genotype more susceptible to subsequent events associated with fatal outcome.

The cause of adverse late events was not recorded in the present study design, and remains to be further clarified in follow-up studies. Of interest, however, is the observation that the survival of patients carrying the deleterious genotype never reached a constant plateau but decreased throughout the follow-up period – which could be explained by the patients being rendered partly immunocompromised during the initial SIRS insult, leading to an increased morbidity and mortality in the period after discharge from the hospital. The small study size probably conveyed insufficient statistical power for significance to be reached before the end of the follow-up. The importance of the near-significant association between 982C>T heterozygosity and early mortality (day 28) was supported by a significantly increased mortality due to infectious causes, and by a consistent association with disease severity parameters predictive of lower survival – such as an increased number of SIRS criteria, a tendency towards an increased SAPS II score and a lower PaO_2_/FiO_2 _ratio [31]. The relatively low number of patients included in the present study is an obvious weakness, calling for further studies to confirm to what extent the genetic polymorphisms in the *HMGB1 *gene can be utilised as severity markers in SIRS and sepsis.

Several clinical studies have been published to date with data on serum HMGB1 levels in various inflammatory settings. Although the studies are heterogeneous in design and population size, a common finding is that patients with infection and/or SIRS have elevated levels of serum HMGB1 as compared with healthy control individuals [9,12-17,32,33]. Conflicting results have been published, however, regarding the correlation between serum HMGB1 levels and disease etiology, severity and mortality, with only a few studies observing a significant increase in serum HMGB1 levels in nonsurvivors with sepsis as compared with survivors [9,32]. Furthermore, only one study found a marginally significant difference in serum levels between infected and noninfected patients with SIRS [15]. The majority of reports do not show a correlation between disease severity and serum HMGB1 levels [12,15,17,32].

Our study is consistent with most of these findings, with lower serum HMGB1 in healthy control individuals as compared with patients, no difference in serum HMGB1 levels according to disease severity, and no correlation with number of SIRS criteria, SAPS II score and PaO_2_/FiO_2 _ratio – although a tendency towards increased levels was observed in patients with sepsis, irrespective of severity, as compared with patients with SIRS without infection. While no association was found according to the 1377delA genotype, the 982C>G genotype was significantly associated with serum HMGB1 levels. Of particular interest in this regard was that HMGB1 could not be detected in 20 healthy control individuals, showing that that the phenotype effect of the *HMGB1 *polymorphism on the HMGB1 serum concentration may only become apparent in disease settings. The finding of lower HMGB1 levels in patients with a higher risk of mortality during their early disease course was surprising, but is in line with reports where nonsignificant trends towards lower serum levels were observed in patients with septic shock and severe sepsis, as compared with patients with less severe disease [12,15]. Furthermore, in a recent study of sepsis, severe cardiovascular failure was significantly associated with lower levels of serum HMGB1 [33]. These findings support the concept of immune paresis, suggesting a primary rather than secondary hypoimmune response, where survival among sepsis patients is associated with the recovery of the inflammatory system and not of the anti-inflammatory system [34].

The conflicting results observed across studies could be explained by additional factors other than the heterogeneity of study populations. HMGB1 acts as a late mediator of sepsis, detectable in septic mice after 8 hours and reaching peak levels after 16 hours [9]. In clinical studies where HMGB1 levels were measured at different time points, two studies report increasing levels during admission [12,17] and one study reports stable levels that persist throughout the first week and after discharge [32]. This opens up the possibility that study patients were either sampled too early or too late in their disease course. In the present study population, the SIRS and sepsis diagnoses were established during the initial 24 hours in the ICU, making it possible that infected patients where diagnosed too early in their disease course to be correctly identified as SIRS with infection. When comparing serum HMGB1 levels across the studies, a striking difference is observed. In studies using ELISA methods [15-17], as in the present study, much lower serum levels are observed compared with studies using blotting techniques [9,12,32]. This discrepancy could be responsible for some of the conflicting results, since it is not known whether the current methods detect different subsets of the protein. Passively released HMGB1 and actively secreted HMGB1 differ at the molecular level [35], and it has been suggested that HMGB1 biological activities could vary depending on these differences [36].

In the present study a total 10 genetic variants were identified, eight of which were classified as polymorphisms and two as mutations. Out of the eight genetic variants classified as polymorphisms, frequency data on seven of the variants have previously been published [23]. The novel 1747delT polymorphism was primarily observed by sequencing in the SIRS population, and was confirmed upon reanalysis in the control population. The frequencies of the polymorphisms in the SIRS population and in the control population were similar (Table 2). The observed slight but significant variation in the distribution of inferred haplotypes between the populations could be attributed to the population sizes, and was without significant impact on disease parameters or outcome.

In accordance with the high degree of HMGB1 structural conservation and with the location of the majority of previously identified genetic variants [23], the novel 1808C>G mutation was located in a noncoding region (Figure 1). By contrast, the novel 4519_4521delGAT mutation was located in a coding region of the gene, and entails a structural change; namely, the deletion of an aspartate amino acid residue from the acidic tail of the finished HMGB1 protein. Previous reports show that both the structure-selective binding of HMGB1 to DNA [37,38] and its interaction with various transcriptional factors [39,40] are highly dependent on the length of the acidic tail. Apart from changing the inherent DNA binding properties of the protein, therefore, the deletion could also cause changes in acetylation and thereby the premise for its release and biological properties [35,36].

## Conclusion

The present study is the first report on the implications of the genetic variation in the human *HMGB1 *gene in a population of critically ill patients admitted to an ICU with SIRS and sepsis. Genetically determined risk factors associated with early and late mortality and death due to infection were revealed, explaining some of the inherited risk in this heterogeneous patient population. Associations between genetic variation and disease severity parameters were also established. Further studies, both clinical and experimental, are therefore needed to confirm the significance of these findings and to explain their molecular background.

## Key messages

• The -1377delA promoter polymorphism was significantly associated with increased risk of delayed mortality in patients with SIRS.

• The 982C>T exon 4 polymorphism was associated with significantly lower levels of serum HMGB1, and with significantly higher probability of early death due to infection.

• The present article is the first report of clinical implications of variation in the human *HMGB1 *gene.

## Abbreviations

ELISA = enzyme-linked immunosorbent assay; HMGB1 = high mobility group box 1 protein; ICU = intensive care unit; PaO_2_/FiO_2 _= ratio between partial pressure of oxygen and fraction of inspired oxygen; PCR = polymerase chain reaction; rs = reference single nucleotide polymorphism number; SAPS II = Simplified Acute Physiology Score II; SIRS = systemic inflammatory response syndrome; TNF = tumor necrosis factor.

## Competing interests

The authors declare that they have no competing interests.

## Authors' contributions

BK analysed samples and data, and drafted the manuscript. LM-F and HOM were involved in the design and practical aspects of the laboratory analyses. JS planned the study, wrote the protocol, collected samples and revised the manuscript. LV was involved in data analysis and manuscript revision. PG planned the study, wrote the protocol, was involved in the genetic and clinical aspects of data analyses and revised the manuscript. All authors read and approved the final manuscript.
